# Prevalence of Cardio-Embolic Brain Complications in Permanent and Paroxysmal Atrial Fibrillation Patients

**DOI:** 10.3390/healthcare11020175

**Published:** 2023-01-06

**Authors:** Ciprian Ilie Rosca, Abhinav Sharma, Daniel-Dumitru Nisulescu, Gabriela Otiman, Daniel-Marius Duda-Seiman, Stelian Ioan Morariu, Daniel Florin Lighezan, Nilima Rajpal Kundnani

**Affiliations:** 1 Centre for Advanced Research in Cardiovascular Pathology and Hemostasis, Victor Babes University of Medicine and Pharmacy, 300041 Timisoara, Romania; 2 Department of Internal Medicine I—Medical Semiotics I, Victor Babes University of Medicine and Pharmacy, 300041 Timisoara, Romania; 3 Department of Internal Medicine, Municipal Emergency University Hospital, 300079 Timisoara, Romania; 4 Civil Medical Society Dr Rosca, 307405 Teremia Mare, Romania; 5 Department of Cardiology, Discipline of Internal Medicine, Ambulatory Care, Prevention and CV Rehabilitation, Victor Babes University of Medicine and Pharmacy, 300020 Timisoara, Romania; 6 Department of Occupational Medicine, Vasile Goldis University of Arad Faculty of Medicine, 473223 Arad, Romania; 7 Department of Occupational Medicine, Municipal Emergency University Hospital, 310025 Arad, Romania; 8 Institute of Cardiovascular Diseases, 300310 Timisoara, Romania; 9 Department of Functional Sciences, Physiology, Centre of Imuno-Physiology and Biotechnologies (CIFBIOTEH), Victor Babes University of Medicine and Pharmacy, 300041 Timisoara, Romania

**Keywords:** atrial fibrillation, permanent AF, paroxysmal AF, cardioembolic brain damage, stroke, dementia, Parkinson’s disease

## Abstract

Background: Atrial fibrillation (AF) is the most frequent of all cardiac arrhythmias, with an increasing prevalence in the last 20 years. Cardio-embolic brain complications (CEBC) related to AF often occur or recur, even following appropriate treatment. Method: We conducted a retrospective study and analyzed the presence of stroke, dementia, and Parkinson’s disease (PD) in both paroxysmal and permanent AF patients. The records of 1111 consecutive admitted patients with primary diagnosis of AF at the Municipal Emergency University Hospital, Timisoara, between 2015 and 2016 were examined. Statistical analysis was performed on the patients included in the study based on the inclusion and exclusion criteria. Results: A significant statistical difference was noted among the permanent AF group for stroke (48.75% vs. 26.74%, *p* < 0.001) and dementia (10.25% vs. 3.86%, *p* < 0.001) compared to paroxysmal AF patients. Permanent AF patients presented a higher risk of developing stroke, dementia, and PD compared to patients with paroxysmal AF. Meanwhile, male gender and an increase in age showed an increase in the odds of having cardio-embolic brain complications in patients with paroxysmal AF. Conclusion: Based on the results obtained, it can be concluded that the risk of cardio-cerebral embolic complications is greater in permanent AF patients compared to paroxysmal AF cases. Ischemic stroke and dementia are more frequent in the permanent AF group, but analyzing the data regarding the age of onset paroxysmal AF is critical due to the fact that it involves a younger population. Prompt diagnosis and treatment can help significantly in saving stroke patients.

## 1. Background

Atrial fibrillation (AF) is the most frequently found cardiac arrhythmia, with an increasing prevalence in the last 20 years [1,2]. Cardio-embolic brain complications (CEBC) related to AF in real-life patients may occur or recur, even in compliant patients (undergoing treatment) in the presence of an appropriate treatment [3]. In this study, we analyzed the impact of the two types of AF (permanent and paroxysmal) on the prevalence of stroke, dementia, and Parkinson’s disease.

Current guidelines regarding AF management recommend anticoagulant therapy for preventing CEBC in both paroxysmal AF and permanent AF [1] in order to reduce the incidence of CEBC [4]. To date, there are a limited number of studies regarding the direct comparison between paroxysmal and permanent AF, and regarding their direct complications where the brain is concerned.

Occurrence or recurrence of stroke depends on AF types and regional income [5,6,7]. In paroxysmal AF, recurrence is rather non-AF related compared to permanent AF [8,9] or persistent AF [3,9,10,11]. These findings remain valid, even in patients with no anticoagulant therapy, but with a higher incidence [12]. In a one-year survey, another study found no difference in the incidence of stroke between paroxysmal AF and permanent AF [13].

On the other hand, it seems that permanent AF patients have mild cognitive deterioration and dementia compared with different types of AF, regardless of stroke presence, but the data are not so consistent [14,15]. Permanent AF is associated with lower Mini-Mental State Examination scores (MMSE) and increased cognitive deterioration [16,17,18], and the risk of dementia is higher regardless of the presence of stroke in these patients [19,20]. Meanwhile, in patients with paroxysmal AF compared to patients with no AF, cognitive impairment and dementia seem to be higher, and they occur at a younger age [21].

In the case of Parkinson’s disease (PD), data are mixed. Studies suggest the linkage of cardiovascular risk factors (including AF) with PD, but in a small proportion compared with dementia [22]. Furthermore, several studies have found a link between PD and the risk of AF development, especially in younger populations, and promote AF as a comorbid association of early PD [23,24].

A major benefit of an earlier diagnosis of AF is to delay the development of cardio-embolic brain complications, mainly because of the comorbid association and interrelation in different proportions of AF, cognitive impairment, dementia, and PD [25,26,27,28].

## 2. Material and Methods

This retrospective study was conducted at the 1st Medical unit of the Municipal Emergency Hospital, Timisoara. We analyzed the patients admitted consecutively with AF diagnostic during the time period of January 2015–December 2016.

A total of 1111 cases were analyzed regarding the presence of stroke, dementia, and PD after the onset of AF; the type of AF (paroxysmal versus permanent); and the medical therapies that can influence the progression of AF. The presence of stroke, dementia, and PD was noted if the diagnosis was made by a neurologist, psychiatrist, or radiologist (data from both present admission and previous medical evaluation/admissions were included). The patients were divided into two groups, based on the type of AF they were suffering with. The paroxysmal AF group (group 1) comprised 389 patients, while 722 patients had permanent AF (group 2). Patients having AF at the time of hospital admission were included in the study. The exclusion criteria included the following factors: if the patient had asked for a discharge against medical advice (DAMA), had suffered mortality during the hospital stay, or was not having AF and was admitted for other cardiac or non-cardiac pathologies. The demographic and clinical characteristics, except for the cardiovascular comorbidities of the two groups, are shown in Table 1.

Overweight was defined as a body mass index (BMI) between 25 and 29.9, obesity grade I as BMI between 30 and 34.9, grade II as a BMI value between 35 and 39.9, and obesity grade III as a BMI value ≥ 40. Glomerular filtration rate (GFR) was calculated using the 2012 CKD-EPI formula, and chronic kidney diseases were staged according to KDOQI guidelines [29]. Hypertension was graded according to the 2018 Guideline for the Management of Hypertension endorsed by the European Society of Cardiology and the European Society of Hypertension. For heart failure (HF) classification, we used the planimetric ejection fraction (EF) and also clinical staging according to the New York Heart Association (NYHA) heart failure classification based on clinical symptoms [30]. Pulmonary hypertension (PH) was estimated using tricuspid regurgitation and peak systolic artery pressure (PSAP) and classified as mild for PSAP between 30 and 44 mmHg, moderate for PSAP between 45 and 70 mmHg, and severe for PSAP over 70 mmHg [31].

### Statistical Analysis

Statistical analysis was performed using IBM SPSS Statistics version 20.0 software for Windows and MedCalc version 20.015 with a significance of *p* < 0.05. We used descriptive statistics, figures, and tables to summarize our findings. Results for targeted variables are presented using descriptive statistics (mean, standard deviation, range, median, and associated interquartile range) for continuous data (*), and counts with associated percentages for categorical data.

## 3. Results

Patients suffering from paroxysmal AF were younger in comparison to the patients having permanent AF.

It was found that younger patients were more likely to have paroxysmal AF compared with Permanent AF; mean age was 69.07 years for group 1 and 74.05 years for group 2, with a statistical significance of *p* < 0.001 in our study.

Table 1 presents a summary of the main characteristics and disease for all patients included in the statistical analysis, and by AF types; *p* values were obtained with independent samples *t*-test (*) and with Chi-square tests/Fisher’s exact tests (^) for statistical testing between paroxysmal AF and permanent AF patients. Continuous data (*) are summarized as mean (standard deviation), minimum and maximum value, and median and associated quartiles (Q1—25 percentage quartile; Q3—75 percentage quartile). Quartiles were obtained with Turkey’s method. Categorical data are presented as counts (percentages) (Table 1).

**Figure 1:**
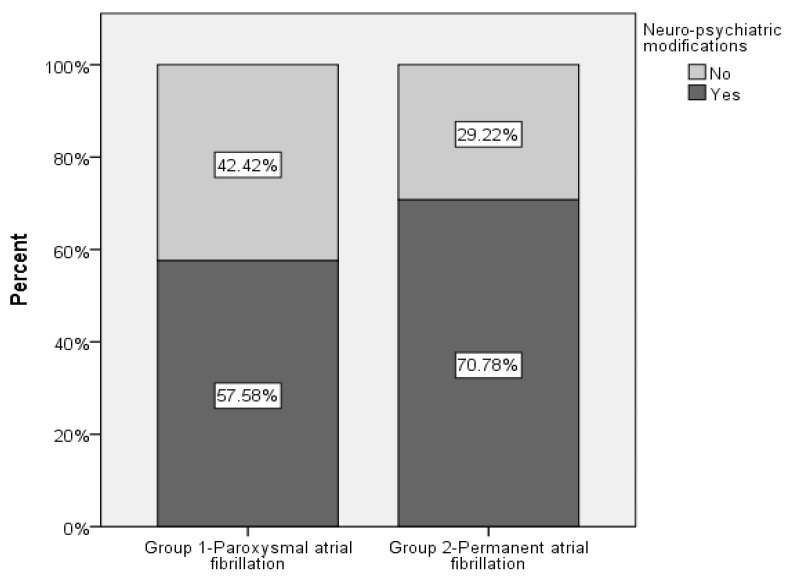
Bar plot with the percentage of patients with neuro-psychiatric changes by AF types.

Neuro-psychiatric changes of any kind were seen in 66.16% of all 1111 investigated patients, irrespective of the time of their first occurrence compared to the date of diagnosis of AF. Details are illustrated in Figure 1.

A significant statistical difference was noted for stroke (48.75% vs. 26.74%, *p* < 0.001) and dementia (10.25% vs. 3.86%, *p* < 0.001) among the two groups. Data regarding stroke, dementia, and Parkinson’s disease are presented in Table 2.

Through the logistic regression analysis, we obtained an increased risk of stroke after the diagnosis of AF for patients with paroxysmal AF and advanced age. The sex and the environment of origin of the patients were not statistically significant in the model, so they were not taken further in the analysis. In addition, the stage of chronic kidney disease had no statistical significance for Parkinson’s disease, dementia, or stroke in the statistical regression analysis, so it was also removed from the final analysis. However, an increased risk of cognitive decline with a factor of 1.966 was detected for patients with BCR of at least stage 3 KDIGO (OR = 1.996, 95% CI 1.149;3.465).

Obesity per se is a risk factor in the occurrence of stroke in patients with paroxysmal atrial fibrillation, but not for patients with permanent atrial fibrillation (Table 3 and Table 4).

Higher odds for overall neuro-psychiatric changes, stroke, dementia, and PD were observed regarding age and paroxysmal AF and are represented in Table 5, Table 6, Table 7 and Table 8. The male gender represents a risk factor for overall neuro-psychiatric changes that cannot be related to the occurrence of stroke, dementia, and PD.

**Figure 2:**
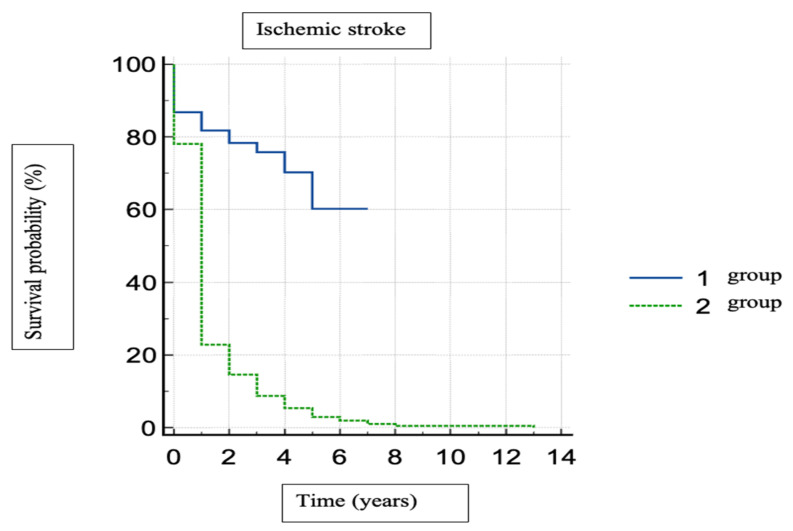
Kaplan–Meier survival probability without stroke. (Group 1—paroxysmal AF, group 2—permanent AF).

Regarding the survival without ischemic stroke after AF onset, group 1 showed a longer period of time. No cerebral cardioembolic complications and the mean period of time with no ischemic stroke was 5.1 years for group 1 and 1.3 years for group 2 (Figure 2).

We noted that ischemic stroke incidence increased with the increase in CHA_2_DS_2_-VASc scores, with scores of 5 and 6 being prominent in stroke patients. Furthermore, even for the acute ischemic stroke and concomitant AF first diagnosis, the mean CHA_2_DS_2_-VASc score for the patients was 5.67 (95% CI [5.3093, 6.0478]) for paroxysmal AF and 5.59 (95% CI [5.3182, 5.8713]) for permanent AF.

The use of OAC treatment is statistically significant in terms of the occurrence of cerebral cardio-embolic complications to the detriment of patients with permanent atrial fibrillation. Thus, overall neuropsychological complications appear statistically significant in the group of patients with permanent AF (*p* = 0.000088), stoke (*p* < 0.00001), and dementia (*p* = 0.014293). Also statistically significant is the presence of stroke (*p* < 0.00001) and dementia (*p* = 0.014146) patients not treated with OAC belonging to the group of patients with permanent AF (Table 9 and Table 10).

For ischemic stroke onset before AF diagnosis (Table 11), the mean age is statistically significant for paroxysmal AF patients (72.6 vs. 76.1 years, *p* < 0.0489 with 95% CI [0.018, 7.35]). Regarding age differences between our two groups for concomitant diagnosis of acute ischemic stroke and first AF, age differences is statistically significant; the paroxysmal AF group has a younger age of 67.8 years vs. 72.5 years (*p* = 0.0084, 95% CI [8.139, 1.206]).

Patients with paroxysmal AF had 1.900 times the odds in comparison to group 2 permanent AF patients of having dementia (OR = 1.900, 95% CI [0.992, 3.641]) (Table 10).

**Figure 3:**
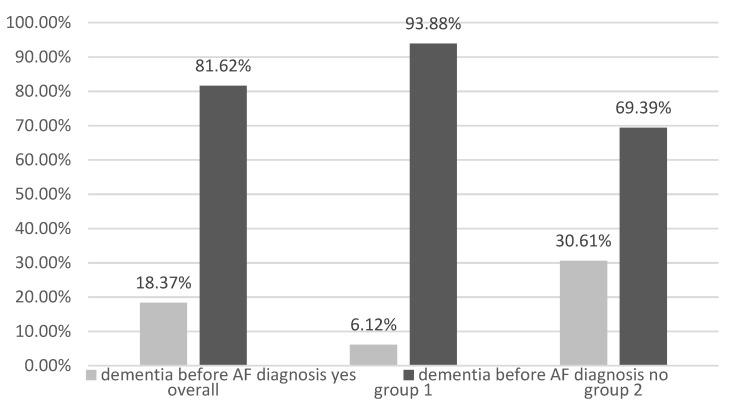
Incidence of dementia before AF diagnosis (AF—atrial fibrillation, group 1—paroxysmal AF, group 2—permanent AF).

**Figure 4:**
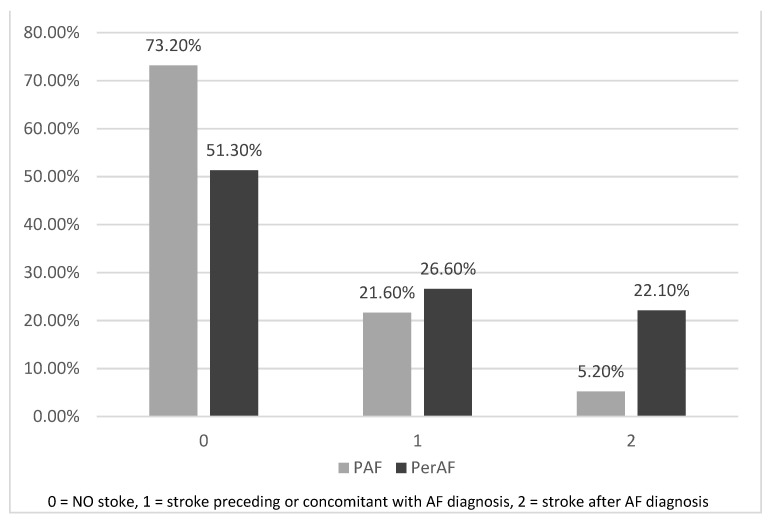
Stoke’s repartition regarding onset time related to the AF diagnosis (PAF—paroxysmal atrial fibrillation, PerAF—permanent atrial fibrillation).

Analyzing the correlation between the onset of AF before stroke, dementia, and PD, we found that dementia is diagnosed before AF diagnosis, and a higher prevalence is observed in the permanent AF group compared to group 1 (Figure 2, Figure 3 and Figure 4).

Even if the presence of stroke is significant for the group of permanent AF patients, when we analyzed its occurrence before the diagnosis of atrial fibrillation, we did not find any statistical difference.

## 4. Discussion

The majority of the studies performed to date are based on identifying the differences between non-AF and AF patients and their related aspects, while we focused on patients having AF of different types (paroxysmal and permanent). Furthermore, we analyzed the prevalence of complications, stroke, dementia, and Parkinson’s disease, seen in both forms of AF, and whether there existed a difference or not.

Our study revealed that the risk of having stroke, dementia, and PD after the onset of paroxysmal AF is more significant than in permanent AF. Male gender and advanced age increase the odds of cardio-embolic brain complications in patients with paroxysmal AF compared to permanent AF.

The prevalence of paroxysmal AF and permanent AF in our study was more significant in women (52.12%, respectively 53.46%) than in men, contradicting actual data [1,32], where it was found to be more prevalent in male patients. One of the most plausible explanations is that, in our country, female to male ratio is higher and women have higher life expectancy compared to males [33]. Furthermore, significant comorbidities are more likely to be present in women than men. Only for paroxysmal AF was the incidence slightly higher in men (50.39%), but without statistical significance.

In our analyses, the relative risk of developing ischemic stroke was comparable with data from the literature [1,34], but this risk was evaluated between the permanent AF group in comparison with paroxysmal AF, and in this manner we were able to more accurately assess the relative risk of ischemic stroke.

Age is a significant determinant of AF type. Older patients are more likely to have permanent AF (*p* < 0.001) than younger patients who have more frequently paroxysmal AF.

In the years 2015–2016, life expectancy in Romania was on average of 75 years [35]. The mean age of patients who developed paroxysmal atrial fibrillation is statistically significantly lower than that of patients who were diagnosed with permanent atrial fibrillation (Table 1). The youngest age at which there was a diagnosis of paroxysmal atrial fibrillation was 26 years compared to the age of 41 years for the youngest patient from the permanent atrial fibrillation group.

The CHA_2_DS_2_-VASc score is a useful tool for estimating the risk of occurrence of ischemic stroke, but it has its own flaws. As seen in our analysis, the highest incidence of ischemic stroke was for middle scores, and the lowest incidence was noted for scores of 8 and 9, which contradicts the findings of previously published studies, which state that the risk of developing ischemic stroke increases with the increase in CHA_2_DS_2_-VASc score [1]. Furthermore, even for acute ischemic stroke and concomitant AF first diagnosis, the mean CHA_2_DS_2_-VASc score for those patients is 5.67 (95% CI [5.3093, 6.0478]) for paroxysmal AF and 5.59 (95% CI [5.3182, 5.8713]) for permanent AF. Ischemic stroke occurs after AF diagnosis for a higher CHA_2_DS_2_-VASc score when we compared it with CHA_2_DS_2_-VASc score for patients with concomitant acute ischemic stroke and AF diagnosis. A large majority of current studies have shown us the inaccuracy of the CHA_2_DS_2_-VASc score in stroke prediction in AF patients [36]

The increase in the overall incidence of neuro-psychological complications, stroke, and dementia among patients with permanent FiA can also be attributed to the more important comorbidities present in these patients. The result obtained from the statistical processing of patients with OAC in terms of Parkinson’s disease is not significant, but the value very close to statistical significance requires revalidation on studies with larger groups of patients. There are also studies that have stated that in patients with atrial fibrillation there is a predisposition regarding the occurrence of Parkinson’s disease that seems to be directly proportional to the increase in intra–atrial conduction time [23,25].

Table 9 and Table 10 show a direct comparison of our two groups by the presence or absence of oral anticoagulation for overall neuro-psychological changes, ischemic stroke, dementia, and Parkinson’s diseases. In patients who have already had an ischemic stroke or have dementia, oral anticoagulation therapy can be beneficial even post event, regardless of the population, as the benefits are the same, based on different studies on different populations, and hence should be continued [37].

Anticoagulants alone cannot provide absolute protection in preventing cardio-embolic cerebral stroke complications. Association with lifestyle modification, management of cardiovascular risk factors, patient awareness and education related to their disease, and physical activity within the patient’s capacity can further help achieve better outcomes [38].

Another important aspect is that a large number of patients had a stroke before AF diagnosis, or the diagnosis of AF was made at the same time as the diagnosis of acute stroke and none of those patient were undergoing ACO therapy at that time. For ischemic stroke onset before AF diagnosis, the mean age is statistically significant for paroxysmal AF patients (72.6 vs. 76.1 years, *p* < 0.0489 with 95% CI [0.018, 7.35]). Regarding age differences between our two groups, for concomitant diagnosis of acute ischemic stroke and first AF, age difference is statistically significant; the paroxysmal AF group has a younger age of 67.8 years vs. 72.5 years (*p* = 0.0084, 95% CI [8.139, 1.206]). In Figure 4, we have demonstrated the ischemic stroke based on the onset time. In 2017, Borowsky et al. showed that 156 of 856 patients (nearly one in five) were admitted for acute stroke at the same time as their first diagnosis of AF [39]. We found in the current medical literature that almost 9% and 25% of new stroke patients have their first diagnosis of AF when they present to the ER due to stroke [40,41,42,43]. Similarly, in our study, the first stroke event coincided with placing the diagnosis of AF in 21.6% of paroxysmal AF patients and in 26.6% of permanent AF group patients having stroke.

Our study reveals once again the fact that anticoagulant treatment is underused, including in Romania, the percentage of patients who do not receive anticoagulant treatment, even if they have an indication for it, falling within the percentages mentioned by other published studies [44,45,46,47]. In our study, 73.08% of patients had received oral anticoagulation before their current hospital admission. The reason behind it being either the patient’s refusal to obey the prescribed treatment plan or the physicians fear to avoid having a hemorrhagic event. For two patients, the confirmation on the use of oral anticoagulation could not be established (and was reported as missing data). It would to ideal establish a safe anticoagulant therapy plan for all patients, but comparing to other studies we can say that 2/3 of our patients succeeded in achieving anticoagulation, which is similar to the finding of other similar studies conducted [1,47].

## 5. Limitations of the Study

Because of the retrospective character of this study, further follow-up was not possible beyond the established study period, which could have given a better insight into the long-term effects of AF. Another limitation is that we have not evaluated the risk of this complication related to other risk factors (such as proinflammatory state, medication use, and so on). More in-depth analysis on the reasons behind the neurological complications could help to better understand whether AF is the only culprit or if there are other factors involved in the pathogenesis of stroke, dementia, and PD. There was no control group comprised of similar age groups to see the age related senile neurological changes. 

## 6. Conclusions

Based on our study results, it can be statistically concluded that permanent AF seems to be worse for the brain than paroxysmal AF. Ischemic stroke and dementia are more frequent in the permanent AF group. We consider that paroxysmal AF is worse due to the fact that a higher number of younger patients are found in this group. Public awareness programs should be implemented from time to time to educate the general population of the alarming signs and symptoms that will help them identify the initiation of a cerebral hypoperfusion that will rapidly end up as ischemic stroke, which will help them to understand that they should call the emergency services. Such self-awareness programs can dramatically minimize the mortality and stroke related disability rates caused due to the delay in reaching specialized care. Regarding the medical unit in charge, initiation of anticoagulant therapy should be prompt after evaluating precisely the risk of hemorrhage, but at the same time without avoiding anticoagulant use due purely to a phobia of encountering a hemorrhagic event.

## Figures and Tables

**Table 1:** Demographic and clinical characteristics (without cardiovascular comorbidities) of study groups. (AF—atrial fibrillation, COPD—chronic obstructive pulmonary disease, CKD—chronic kidney disease, GFR—glomerular filtration rate, HDLc—high-density lipoprotein cholesterol, LDLc—low-density lipoprotein cholesterol, Q1—25 percentage quartiles, Q3—75 percentage quartiles, SD—standard deviation, (*)—continuous data).

		Group 1 Paroxysmal AF (N = 389)	Group 2 Permanent AF (N = 722)	*p-*Value
Age, years, mean, (SD) (*)		69.07 (10.950)	74.05 (9.978)	<0.001
Sex	Female	193 (49.61%)	386 (53.46%)	0.221
	Male	196 (50.39%)	336 (46.54%)	
Urban vs. rural	Urban (city/town)	226 (58.10%)	335 (46.40%)	<0.001
	Rural (village)	163 (41.90%)	387 (53.60%)	
Overweight		18 (4.63%)	53 (7.34%)	0.078
Obesity	Overall	107 (27.51%)	178 (24.65%)	0.299
	Grade I	57 (14.7%)	97 (13.4%)	0.5640
	Grade II	34 (8.8%)	40 (5.5%)	0.0402
	Grade III	16 (4.1%)	41 (5.7%)	0.2672
CKD stage	1	41 (10.54%)	42 (5.82%)	<0.001
	2	139 (35.73%)	204 (28.25%)	
	3a	104 (26.74%)	179 (24.79%)	
	3b	71 (18.25%)	194 (26.87%)	
	4	24 (6.17%)	71 (9.83%)	
	5	8 (2.06%)	27 (3.74%)	
	Missing/Unknown	2 (0.51%)	5 (0.69%)	
GFR, (*) mean; (SD)		58.806; (22.6158)	52.651; (22.175)	<0.001
Diabetes mellitus		128 (32.9%)	209 (28.95%)	0.171
COPD		81 (20.82%)	156 (21.61%)	0.761
Asthma		41 (10.54%)	72 (9.97%)	0.765
Hyperuricemia		47% (12.08%)	112 (15.51%)	0.119
Dyslipidemia		278 (71.47%)	446 (61.77%)	0.001
High LDLc levels Missing/Unknown		222 (57.07%) 1 (0.26%)	379 (52.49%) 1 (0.14%)	0.138
Low HDLc levels Missing/Unknown		122 (31.36%) 1 (0.26%)	228 (31.58) 1 (0.14%)	0.951
Hypercholesterolemia Missing/Unknown		269 (69.15%) 1 (0.26%)	440 (60.94%) 1 (0.14%)	0.006
Hypertriglyceridemia Missing/Unknown		189 (48.59%) 1 (0.26)	325 (45.01%) 1 (0.14%)	0.247

**Table 2:** Presence of stoke, dementia, and PD among the two study groups.

	Group 1 Paroxysmal AF (N = 389)	Group 2 Permanent AF (N = 722)	*p-*Value
Stroke Missing/Unknown	104 (26.74%) 1 (0.26%)	351 (48.75%) 1 (0.14%)	<0.001
Dementia	15 (3.86%)	74 (10.25%)	<0.001
Parkinson’s disease	14 (3.6%)	43 (5.96%)	0.089

**Table 3:** Logistic regression analysis (Enter method) taking stroke as a dependent variable for patients with paroxysmal AF for overweight and obesity.

Variables in the Equation	B	S.E.	Wald	df	Sig.	Exp(B)	95% C.I. for EXP(B)	
							Lower	Upper
Overweight	−0.674	0.600	1.261	1	0.261	0.510	0.157	1.652
Obesity	1.219	0.635	3.684	1	0.045	3.383	1.975	11.746
Constant	−2.331	1.251	3.471	1	0.062	0.097		

**Table 4:** Logistic regression analysis (Enter method) taking stroke as a dependent variable for patients with permanent AF for overweight and obesity.

Variables in the Equation	B	S.E.	Wald	df	Sig.	Exp(B)	95% C.I. for EXP(B)	
							Lower	Upper
Overweight	0.144	0.308	0.217	1	0.642	1.154	0.631	2.113
Obesity	0.464	0.335	1.915	1	0.166	1.590	0.825	3.065
Constant	−0.237	0.762	0.096	1	0.756	0.789		

**Table 5:** Logistic regression analysis for occurrence risk of neuropsychiatric changes, stroke, dementia, after AF diagnosis.

	Group 1 Paroxysmal AF N (%) *	Group 2 Permanent AF N (%) *	OR (95% CI) Group 1 Group 2	*p-*Value
Neuro-psychiatric changes	298 (26.82%)	598 (53.65%)	1.848 (1.373; 2.486) 1	<0.001
Stroke	49 (4.41%)	266 (23.94%)	3.773 (2.662; 5.348) 1	<0.001
Dementia	12 (1.08%)	59 (5.31%)	1.900 (0.992; 3.641) 1	0.053

AF—atrial fibrillation, N—number of patients, OR—odds ratio, 95% CI—95% confidence interval, *—percentage based on the total number of patients (1111 patients).

**Table 6:** Multiple logistic regression analysis for risk of neuro-psychiatric changes after AF diagnosis for AF type, gender, and age.

	Patients with Neuro-Psychiatric Changes N (%) *	OR (95% CI)	*p*-Value
AF type Group 1—Paroxysmal AF Group 2—Permanent AF	298 (26.82%) 596 (53.65%)	1.848 (1.373; 2.486) 1	<0.001
Gender Female Male	447 (40.23%) 447 (40.23%)	1 1.657 (1.250; 2.197)	<0.001
Age (years)	894 (80.47%)	1.041 (1.027; 1.056)	<0.001
Urban (city/town) Rural (village)	452 (40,68%) 442 (39.78%)	0.8371 (0.5194; 1.3492)	0.4653
CKD	894 (80.47%)	1.0544 (0.8460; 1.3141)	0.6371
Dyslipidemia	894 (80.47%)	1.2068 (0.6320; 2.3045)	0.5690
Hypercholesterolemia	894 (80.47%)	0.7352 (0.3820; 1.4149)	0.3570

AF—atrial fibrillation, CKD—chronic kidney disease, N—number of patients, OR—odds ratio, 95% CI—95% confidence interval, *—percentage based on the total number of patients (1111 patients).

**Table 7:** Multiple logistic regression analysis for risk of stroke after AF diagnosis for AF type and age.

	Patients with Stroke N (%) *	OR (95% CI)	*p*-Value
FIA type Group 1—Paroxysmal AF Group 2—Permanent AF	49 (4.41%) 266 (23.94%)	3.773 (2.662; 5.348) 1	<0.001
Age (years)	315 (28.35%)	1.029 (1.014; 1.044)	<0.001

AF—atrial fibrillation, N—number of patients, OR—odds ratio, 95% CI—95% confidence interval, *—percentage based on the total number of patients (1111 patients).

**Table 8:** Multiple logistic regression analysis for risk of dementia after AF diagnosis for AF type and age, AF—atrial fibrillation, N—number of patients, OR—odds ratio, 95% CI—95% confidence interval, *—percentage based on the total number of patients (1111 patients).

	Patients with Stroke N (%) *	OR (95% CI)	*p*-Value
FIA type Group 1—Paroxysmal AF Group 2—Permanent AF	12 (1.08%) 59 (5.31%)	1.900 (0.992; 3.641) 1	<0.053
Age (years)	71 (6.39%)	1.088 (1.056; 1.122)	<0.001

**Table 9:** Comparing patients with paroxysmal AF and permanent AF in the prevention of overall neuropsychiatric complications, stroke, dementia, and Parkinson’s disease undergoing treatment with and without oral anticoagulants, (using the Chi-square test).

		With OAC			Without OAC		
		Group 1	Group 2	*p*	Group 1	Group 2	*p*
NS	no	N = 64(38.68%)	N = 134(29.37%)	*p* = 0.000088	N = 100(43.82%)	N = 78(29.23%)	*p* = 0.308255
	yes	N = 85(61.32%)	N = 377(70.63%)		N = 140(56.18%)	N = 133(70.77%)	
Stroke	no	N = 107(75.18%)	N = 259(44.37%)	*p* = <0.00001	N = 178(72.11%)	N = 112(53.30%)	*p* < 0.00001
	yes	N = 42(24.82%)	N = 252(55.63%)		N = 62(27.89%)	N = 99(46.70%)	
Dementia	no	N = 147(93.43%)	N = 462(85.62%)	*p* = 0.014293	N = 227(97.60%)	N = 186(91.08%)	*p* = 0.014146
	yes	N = 2(6.57%)	N = 33(14.38%)		N = 13(2.30%)	N = 25(8.92%)	
Parkinson’s disease	no	N = 145(95.62%)	N = 478(94.37%)	*p* = 0.078101	N = 230(96.81%)	N = 201(93.93%)	*p* = 0.768168
	yes	N = 4(4.38%)	N = 33(5.63%)		N = 10(3.19%)	N = 10(6.07%)	

(NS—neurological complications, OAC—oral anticoagulants).

**Table 10:** Impact of oral anticoagulants in the appearance of overall neuro-psychiatric complications, stroke, dementia, and Parkinson’s disease in paroxysmal and permanent AF patients (using Chi-square test).

		Group 1 with OAC	Group 1 without OAC	*p*	Group 2 with OAC	Group 2 without OAC	*p*
NS	no	N = 64(38.68%)	N = 100(43.82%)	*p* = 0.802774	N = 134(29.37%)	N = 78(29.23%)	*p* = 0.003941
	yes	N = 85(61.32%)	N = 140(56.18%)		N = 377(70.63%)	N = 133(70.77%)	
Stroke	no	N = 107(75.18%)	N = 178(72.11%)	*p* = 0.609987	N = 259(44.37%)	N = 112(53.30%)	*p* = 0.558054
	yes	N = 42(24.82%)	N = 62(27.89%)		N = 252(55.63%)	N = 99(46.70%)	
Dementia	no	N = 147(93.43%)	N = 227(97.60%)	*p* = 0.042472	N = 462(85.62%)	N = 186(91.08%)	*p* < 0.00001
	yes	N = 2(6.57%)	N = 13(2.30%)		N = 33(14.38%)	N = 25(8.92%)	
Parkinson’s disease	no	N = 145(95.62%)	N = 230(96.81%)	*p* = *0*.445518	N = 478(94.37%)	N = 201(93.93%)	*p* = 0.374859
	yes	N = 4(4.38%)	N = 10(3.19%)		N = 33(5.63%)	N = 10(6.07%)	

(NS—neurological complications, OAC—oral anticoagulants).

**Table 11:** Presence of stroke in the two study groups and its presence before the diagnosis of AF.

	Total Population (N = 1111) (N * = 167)	Group 1—Paroxysmal AF (N = 389) (N * = 63)	Group 2—Permanent AF (N = 722) (N * = 104)	*p*-Value
Stroke No Yes Missing/Unknown	654 (58.87%) 455 (40.95%) 2 (0.18%)	284 (73.01%) 104 (26.74%) 1 (0.26)	370 (51.25%) 351 (48.75%) 1 (0.14%)	*p* < 0.001
Stroke before AF diagnosis No Yes	27 (16.17%) 140 (83.83%)	8 (12.70%) 55 (87.30%)	19 (18.27%) 85 (81.73%)	*p* = 0.343

AF—atrial fibrillation, * patients with stroke before AF diagnosis.

## Data Availability

Data will be provided on written request.
